# *Coxiella* Endosymbiont of *Rhipicephalus microplus* Modulates Tick Physiology With a Major Impact in Blood Feeding Capacity

**DOI:** 10.3389/fmicb.2022.868575

**Published:** 2022-05-03

**Authors:** Melina Garcia Guizzo, Lucas Tirloni, Sergio A. Gonzalez, Marisa D. Farber, Glória Braz, Luís Fernando Parizi, Lucas Andre Dedavid e Silva, Itabajara da Silva Vaz, Pedro L. Oliveira

**Affiliations:** ^1^Vector Biology Section, Laboratory of Malaria and Vector Research, National Institute of Allergy and Infectious Diseases, Rockville, MD, United States; ^2^Instituto de Bioquímica Médica Leopoldo de Meis, Universidade Federal do Rio de Janeiro, Rio de Janeiro, Brazil; ^3^Tick-Pathogen Transmission Unit, Laboratory of Bacteriology, Rocky Mountain Laboratories, National Institute of Allergy and Infectious Diseases, Hamilton, MT, United States; ^4^Instituto de Agrobiotecnologia y Biologia Molecular (IABIMO), INTA-CONICET, Hurlingham, Argentina; ^5^Instituto de Química, Universidade Federal do Rio de Janeiro, Rio de Janeiro, Brazil; ^6^Centro de Biotecnologia, Universidade Federal do Rio Grande do Sul, Porto Alegre, Brazil; ^7^Faculdade de Veterinária, Universidade Federal do Rio Grande do Sul, Porto Alegre, Brazil; ^8^Instituto Nacional de Ciência e Tecnologia em Entomologia Molecular (INCT-EM), Rio de Janeiro, Brazil

**Keywords:** *Coxiella*, symbiont, *Rhipicephalus microplus*, transcriptome, microbiome, tick

## Abstract

In the past decade, metagenomics studies exploring tick microbiota have revealed widespread interactions between bacteria and arthropods, including symbiotic interactions. Functional studies showed that obligate endosymbionts contribute to tick biology, affecting reproductive fitness and molting. Understanding the molecular basis of the interaction between ticks and their mutualist endosymbionts may help to develop control methods based on microbiome manipulation. Previously, we showed that *Rhipicephalus microplus* larvae with reduced levels of *Coxiella* endosymbiont of *R. microplus* (CERM) were arrested at the metanymph life stage (partially engorged nymph) and did not molt into adults. In this study, we performed a transcriptomic differential analysis of the *R. microplus* metanymph in the presence and absence of its mutualist endosymbiont. The lack of CERM resulted in an altered expression profile of transcripts from several functional categories. Gene products such as DA-P36, protease inhibitors, metalloproteases, and evasins, which are involved in blood feeding capacity, were underexpressed in CERM-free metanymphs. Disregulation in genes related to extracellular matrix remodeling was also observed in the absence of the symbiont. Taken together, the observed alterations in gene expression may explain the blockage of development at the metanymph stage and reveal a novel physiological aspect of the symbiont-tick-vertebrate host interaction.

## Introduction

Ticks are blood-sucking arthropods that parasitize a wide range of hosts. They harbor a variety of microorganisms, such as bacteria, viruses, and protozoa, being one of the main vectors of pathogenic agents that cause human and veterinary diseases (Jongejan and Uilenberg, 2004).

In the past decade, metagenomic studies using high throughput sequencing have explored the overall microbial diversity of ticks (Andreotti et al., 2011; Bonnet et al., 2017; Greay et al., 2018; Maldonado-Ruiz et al., 2021). Several tick-endosymbiont associations have been identified and some have been functionally characterized (Zhong et al., 2007; Guizzo et al., 2017; Zhang et al., 2017; Duron et al., 2018; Ben-Yosef et al., 2020). Studies revealed that the genomes of ovarian endosymbionts encode genes involved in the synthesis of B vitamins and cofactors (Hunter et al., 2015; Gerhart et al., 2016; Guizzo et al., 2017; Duron et al., 2018). These findings provided a likely molecular basis for the mutualistic features of these relationships.

In metazoans, vitamins are frequently provided by the microbial community residing in the digestive tract (Rowland et al., 2018; Jing et al., 2020). We recently showed, however, that the abundance of the bacterial midgut community in *Rhipicephalus microplus* is extremely low compared to other hematophagous arthropods (Guizzo et al., 2020). Additionally, the *R. microplus* ovarian microbiome mostly consisted of the endosymbiont *Coxiella* sp., which exceeded the midgut 16S rDNA copy numbers by several orders of magnitude. These results suggest that the physiological role exercised by gut bacteria in most metazoans is accomplished in *R. microplus* by this abundant symbiont housed in the ovary, which may supply its tick host with nutrients missing or in low concentrations in the vertebrate host blood.

The *Coxiella* endosymbiont of *R. microplus* (CERM) is vertically transmitted, comprising 98% of the 16S rRNA sequences in eggs and larvae (Guizzo et al., 2017). It has been identified in 100% of the *R. microplus* specimens collected from different locations (Duron et al., 2015). CERM is essential to adult emergence since tetracycline-treatment of the tick caused an interruption in development at the metanymph (partially engorged nymph) stage. Although it is known that non-pathogenic bacteria of *Coxiella* genus are obligate endosymbionts in several tick species and contribute to the tick physiology (Zhong et al., 2007; Machado-Ferreira et al., 2016; Duron et al., 2017; Ben-Yosef et al., 2020; Zhong et al., 2021), the mechanism underlying this mutualistic relationship is still unknown in *R. microplus*.

In order to investigate the functional contribution of CERM to the development of its host, we performed a transcriptomic analysis and evaluated the tick genes modulated in the absence of the symbiont. The results described here reveal an altered expression pattern in several metabolic pathways with a major impact on genes involved in blood feeding capacity, such as DA-P36 family members, protease inhibitors, metalloproteases, and evasins.

## Materials and Methods

### *Rhipicephalus microplus* Strain

*Rhipicephalus microplus* ticks (Porto Alegre strain) are a reference tick population with high susceptibility to synthetic acaricides. Ticks were wild-caught from the rural area of Porto Alegre (Rio Grande do Sul, Brazil), and the colony has been maintained with any addition of ticks from other collections and without exposure to acaricides for more than 30 years. The ticks were fed on Hereford cattle obtained from a naturally tick-free area (Santa Vitória do Palmar, Brazil; 33° 32′2″ S, 53° 20′59” W) and kept in individual tick-proof pens on a slatted floor at the Faculdade de Veterinária of Universidade Federal do Rio Grande do Sul (Brazil; Reck et al., 2009). During experiments, fully engorged females, eggs, and larvae were kept in an incubator at 28°C and 80% relative humidity. Calves were infested with 15-day-old tick larvae. All animal care and experimental protocols were conducted following the guidelines of the institutional care and use committee (Ethics Committee on Animal Experimentation of the Universidade Federal do Rio Grande do Sul) and were approved under registry 28,108 and 14,403.

### Generation of CERM-Free Metanymphs

In a previous study (Guizzo et al., 2017), we showed the generation of *R. microplus* progeny with significantly reduced levels of CERM through the antibiotic treatment. Briefly, fully engorged females (average weight 250 mg/tick) were injected with 1 μl of 7.5 μg/μL tetracycline hydrochloride (Merck, Darmstadt, HE, DE) diluted in 0.15 M NaCl, 10 mM sodium phosphate, pH 7.4 (PBS), or PBS only, using a micro-syringe (Hamilton-33-gauge needle). The larvae resulting from clutches of eggs with significantly reduced levels of CERM or control larvae with normal levels of CERM were used to infest one head of cattle, each group being placed separately into individual cotton bags glued to the host skin. Ticks from both groups were collected in several days during development on host revealing that those from the group with lower levels of CERM stopped feeding and develop on the 14th day post-infestation at the metanymph stage. Here, we described a follow-up of this study through the investigation of the impact of the lack of CERM in *R. microplus’* physiology. To this aim, the RNA of those aposymbiotic metanymphs and their symbiotic counterparts generated previously (Guizzo et al., 2017) were isolated and used in a comparative transcriptomic analysis as described below.

### RNA Isolation and CERM Relative Abundance Analysis

Total RNA was isolated individually from whole metanymphs in control (*n* = 5) and tetracycline-treated (*n* = 5) groups generated in a previous study (Guizzo et al., 2017) using TRIzol (Invitrogen, Carlsbad, CA, United States) according to manufacturer’s instructions. RNA concentration and purity were determined on a NanoDrop spectrophotometer. Complementary DNA (cDNA) was synthesized from equal amounts (~ 1  μg) of total RNA using the High Capacity cDNA Reverse Transcription kit (Applied Biosystems, Waltham, MA, EUA). CERM relative abundance was quantified in metanymph ticks as described by Guizzo et al. (2017), using primers (forward-5′TTCGGTGGGAAAGAAAGTTTC3′; reverse-5′TAGGGCTTTCACATTCGACTTA-AAT3′) specific for the 16S rRNA gene sequence (KT726373) of CERM. *R. microplus* 40 S ribosomal gene (EW679928) was used as a reference gene for data normalization (forward-5′GGACGACCGATGGCTACCT3′; reverse 5′TGAGTTGATTGGCGCACTTCT3′; Pohl et al., 2008). qPCR was carried out on a Rotor-Gene Q (Qiagen, Hilden, DE) with 35 cycles of 95°C (5 s) and 60°C (10 s) following an initial denaturation of 95°C (5 min). A melting curve was generated to confirm the identity of amplicons. Each 25 μl reaction mixture contained 12.5 μl of 2 × Rotor-Gene SYBR Green PCR Master Mix containing AmpliTaq Gold^®^ DNA polymerase (Qiagen, Hilden, DE), 3.5 μM of each primer, and 5 μl of cDNA 10 × diluted in sterile ultrapure water. Relative abundance was analyzed by the comparative Ct method (Pfaffl, 2001). The samples were considered free of CERM if no specific amplification for the bacterium could be detected. The detection of *R. microplus* 40S ribosomal gene in all the tick samples indicated the successful DNA isolation, validating the results of the absence of symbiont by qPCR.

### cDNA Library Construction and NGS Sequencing

Total RNA was isolated as described above from 6 pools of samples, 3 from control, and 3 from tetracycline-treated groups (containing 3 metanymphs each). Final RNA concentration was measured using a Qubit fluorometer (Thermo Fisher Scientific, Carlsbad, CA, United States) and integrity and purity were assessed using an Agilent Bionalyzer 2100 with an RNA 6000 Nano chip (Agilent Technologies, Santa Clara, CA, United States). cDNA libraries for Illumina sequencing were constructed using TruSeq Stranded mRNA kit (Illumina, San Diego, CA, United States). Briefly, the purification of mRNA was performed using oligo-dT beads provided in the kit. The first and second strand of cDNA were synthesized from the purified mRNA. The double-stranded cDNA ends were adenylated and ligated to the adaptor. Library enrichment and ligation of specific Illumina indexes were performed by PCR. The libraries were quantified using KAPA library quantification kit (KAPA Biosystems, Basileia, SWI). The sequencing was performed in an Illumina-Hiseq platform. The six RNA-seq libraries generated a total of 13.4 million paired-end reads (2 × 150 bp).

### Bioinformatic Analysis

Fastq files were trimmed of low-quality reads and contaminating primer sequences were removed using Trim Galore.^1^ The quality-filtered sequence reads of each sample were pooled and assembled with Abyss software with various k values (from 25 to 95 at ten-unit intervals; Simpson et al., 2009). Because the Abyss software tends to miss highly expressed contigs, we utilized the Trinity assembler (Grabherr et al., 2011). The resulting assemblies were joined and contigs smaller than 100 nt and sharing more than 95% identity were removed. Coding sequences (CDS) were extracted using an automated pipeline (Karim et al., 2011), based on the existence of a signal peptide in the longer open reading frame (ORF) and by similarities to other proteins found in the Refseq-invertebrate database and proteins from Chelicerata deposited at the National Center for Biotechnology Information (NCBI). A non-redundant set of the CDS and their protein sequences were mapped into a hyperlinked Excel spreadsheet. Predictions of signal peptide, transmembrane domains, and glycosylation sites were determined with software from the Center for Biological Sequence Analysis.^2^ Automated annotation of the proteins was based on matches to various databases, including Gene Ontology^3^ (Harris et al., 2008), CDD,^4^ KOG^5^ (Tatusov et al., 2000), Refseq-invertebrates, and sequences containing Chelicerata [organism] protein sequences obtained from GenBank,^6^ Uniprot,^7^ and the TickSialoFam (TSFam), a database used for the identification of transcripts from tick salivary glands (Ribeiro and Mans, 2020). Transcripts with coverage >67% matching those from TSFam database were considered from salivary origin. Manual annotation was performed as detailed previously (Karim et al., 2011). To estimate transcript abundance, reads for each library were mapped on the deducted CDS using the RSEM software (Li and Dewey, 2011) and statistical tests were performed using the package edgeR (Empirical analysis of digital gene expression data in R; Robinson et al., 2010). BLASTp searches of extracted protein-encoding sequences against the *R. microplus* genome (Jia et al., 2020) and BUSCO (Seppey and Manni, 2019) analysis using the arthropoda database were used to assess the assembly quality and completeness. The R Bioconductor package edgeR (Robinson et al., 2010) was used to construct a multidimensional scaling plot (MDS) and a MA plot (transforming the data into M (log ratio) and A (mean average) scales), in which all transcripts were included in order to investigate the level of similarity among metanymph samples and visualize the dispersion of the transcripts. The same package was used to identify differentially expressed transcripts (DET) between metanymphs in the presence (CERM) or absence of CERM (CERM-free). Low count transcripts were filtered out following the cut off rule of 10 count per million (CPM) in at least three libraries. The DET were identified using Fisher’s exact test, based on a threshold of FDR < 0.05. The FDR (false discovery rate) was used to control the rate of false positive in multiple testing.

### Data Availability

Raw sequence reads were deposited in the NCBI Sequence Read Archive. Accession number: SRR12551220, SRR12551221, SRR12551222 (normal metanymph) and SRR12551217, SRR12551218, and SRR12551219 (CERM-free metanymph; Biosample SAMN02463642 and Bioproject PRJNA660356). Transcriptome Shotgun Assembly (TSA) project has been deposited at DDBJ/EMBL/GenBank under the accession GJSL00000000. The version described in this paper is the first version, GJSL01000000.

## Results and Discussion

### Antibiotic Treatment Generated CERM-Free Metanymph Ticks

**Figure 1:**
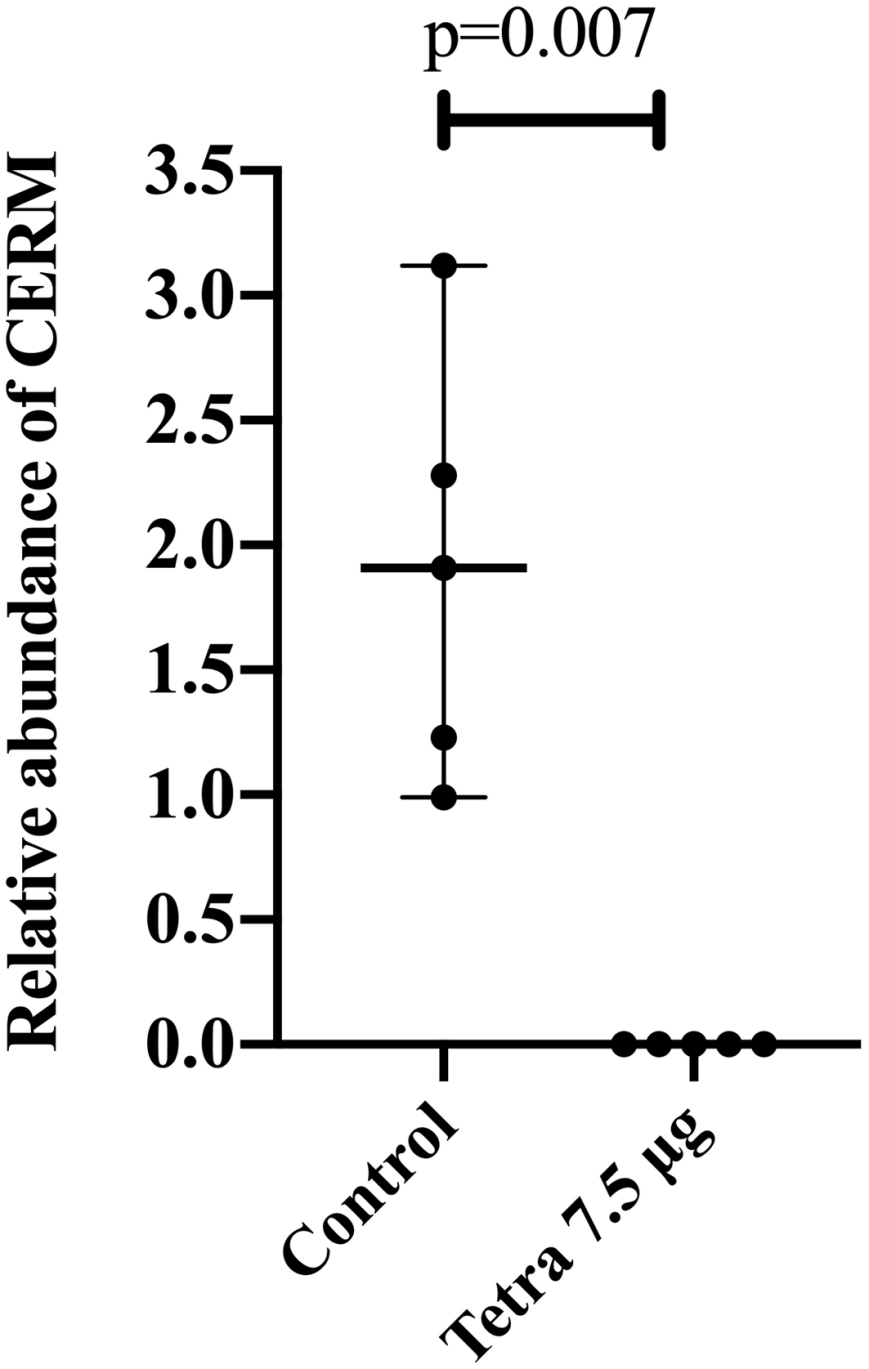
Relative abundance of CERM (*Coxiella* endosymbiont from *Rhipicephalus microplus*) analyzed by qPCR in metanymphs from control and tetracycline-treated groups collected from the host on the 14th post-infestation day. Each dot represents an individual whole metanymph. The levels of CERM were expressed as the median with 95% of CI of five biological samples with three technical replicates in each point. Statistical analyses: Mann–Whitney test.

Tick symbionts are associated with host fitness since their elimination can negatively impact egg laying, egg hatching, and tick growth or survival (Zhong et al., 2007; Guizzo et al., 2017; Zhang et al., 2017; Duron et al., 2018; Ben-Yosef et al., 2020). To date, these functional studies have analyzed the effects of the significantly reducing symbionts on tick fitness. In a previous study, we showed that recently hatched larvae of tetracycline-treated fully engorged females presented in lower abundance of CERM compared to the control group (Guizzo et al., 2017). When these reduced-CERM larvae were allowed to feed on a head of cattle, tick development was blocked in the metanymph life stage (Guizzo et al., 2017). In the present study, we found that the metanymphs that developed from these tetracycline-treated ticks were completely CERM-free (Figure 1). Therefore, one hypothesis is that the bacterial transcripts that were detected by qPCR in the previous report in unfed larvae population (Guizzo et al., 2017) were merely non-viable remnants of the original bacterial. Alternatively, the few surviving bacterial cells were eliminated by larvae after the onset of the blood meal, resulting in the total elimination of the endosymbiont.

This discovery allowed us to analyze tick transcriptional responses in the absence of a vertically inherited symbiont. It has been shown that for other tick symbionts such as *Midichloria mitochondrii* and *Rickettsia buchneri*, bacteria expand during larval and nymphal engorgement phases and decrease after molting into the following life stage (Sassera et al., 2008; Oliver et al., 2021). This fluctuation in bacterial growth during development might also apply to CERM and its coincidence with life stage progression may explain the interruption of development at the metanymph stage. Larvae with low CERM abundance kept feeding and growing, but the symbiont was critical in the nymphal stage since its absence caused a blockage in the development. Alternatively, there may be changes in the relationship between *R. microplus* and CERM depending on the developmental stage of the tick, with nymphs more reliant on CERM than larvae.

### Identification and Analysis of the Differentially Expressed Transcripts

**Figure 2:**
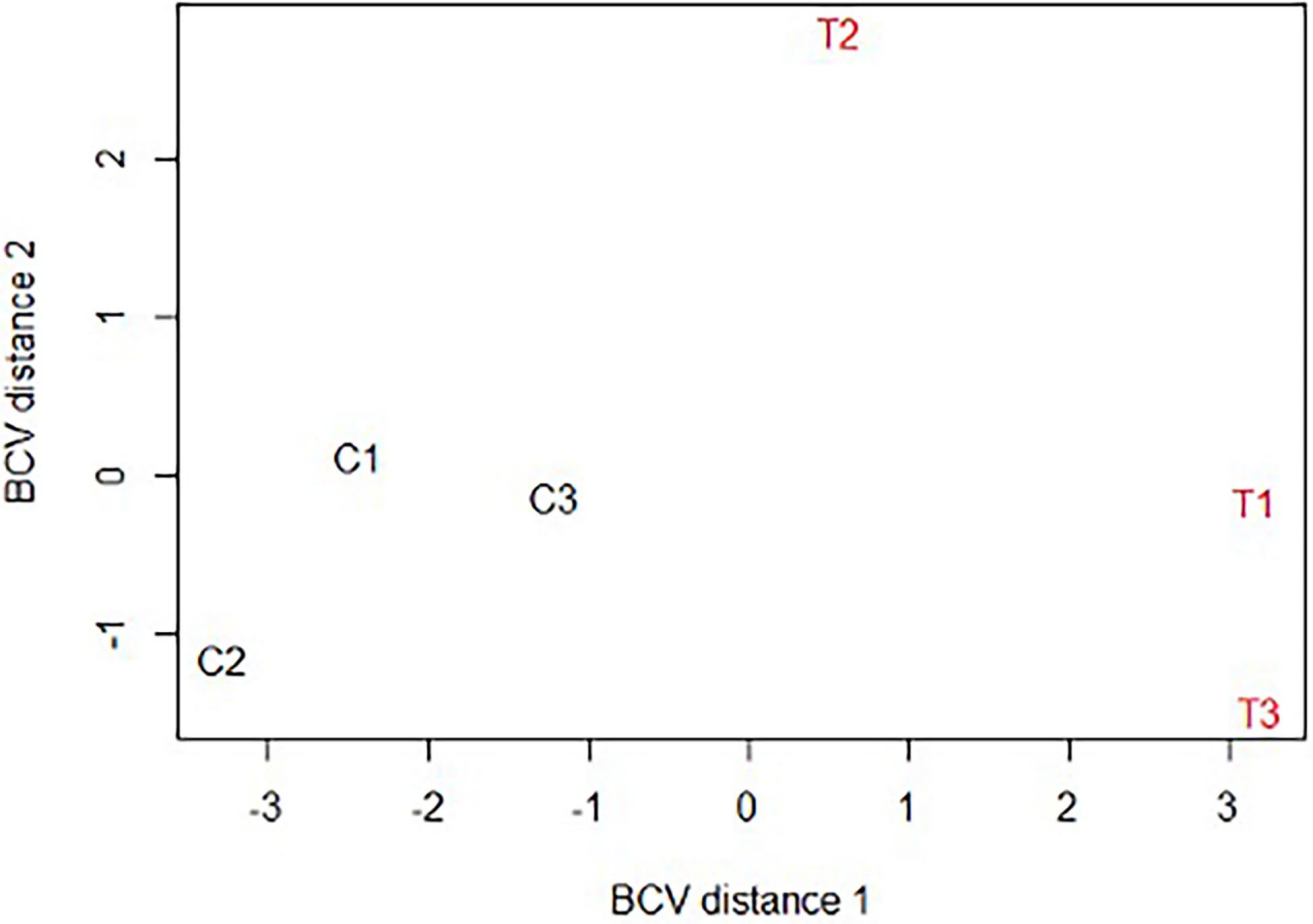
Multidimensional scaling plot (MDS) of *Rhipicephalus microplus* CERM-depleted and control metanymphs. C1, C2, C3: CERM metanymphs; T1, T2, T3: CERM-free metanymphs. BCV, Biological coefficient of variation.

After assembly of the paired-end reads, a total of 775,192 contigs were obtained from which 48,526 coding sequences were extracted and categorized according to biological functions as described in the Materials and Methods section (Supplementary Table 1- worksheet: all transcripts). A BUSCO (Benchmarking Universal Single-Copy Orthologs) analysis of the transcriptome showed a score of 87.7%, indicating a high degree of completeness of the assembly. The multidimensional scaling plot showed clearly distinct clustering of controls separated from CERM-free metanymph samples, suggesting that large changes in gene expression occur in the absence of the symbiont (Figure 2).

**Figure 3:**
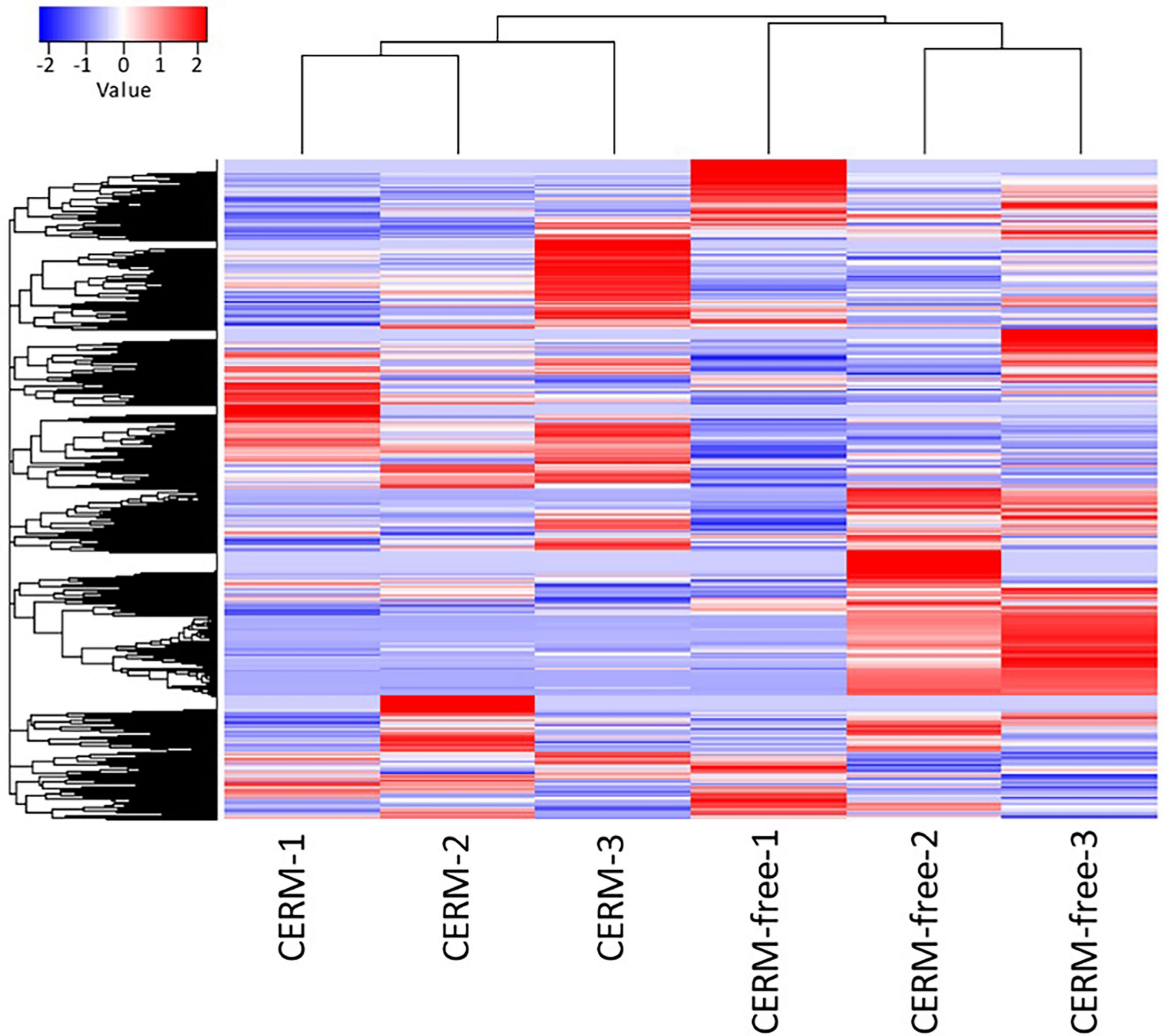
Differentially expressed transcripts in CERM (*Coxiella* endosymbiont from *Rhipicephalus microplus*) and CERM-free metanymphs. Heat map of normalized TPM data for CERM and CERM-free replicates. The row Z-score color scale accounts for the Z-score deviation from the mean by standard deviation units.

**Figure 4:**
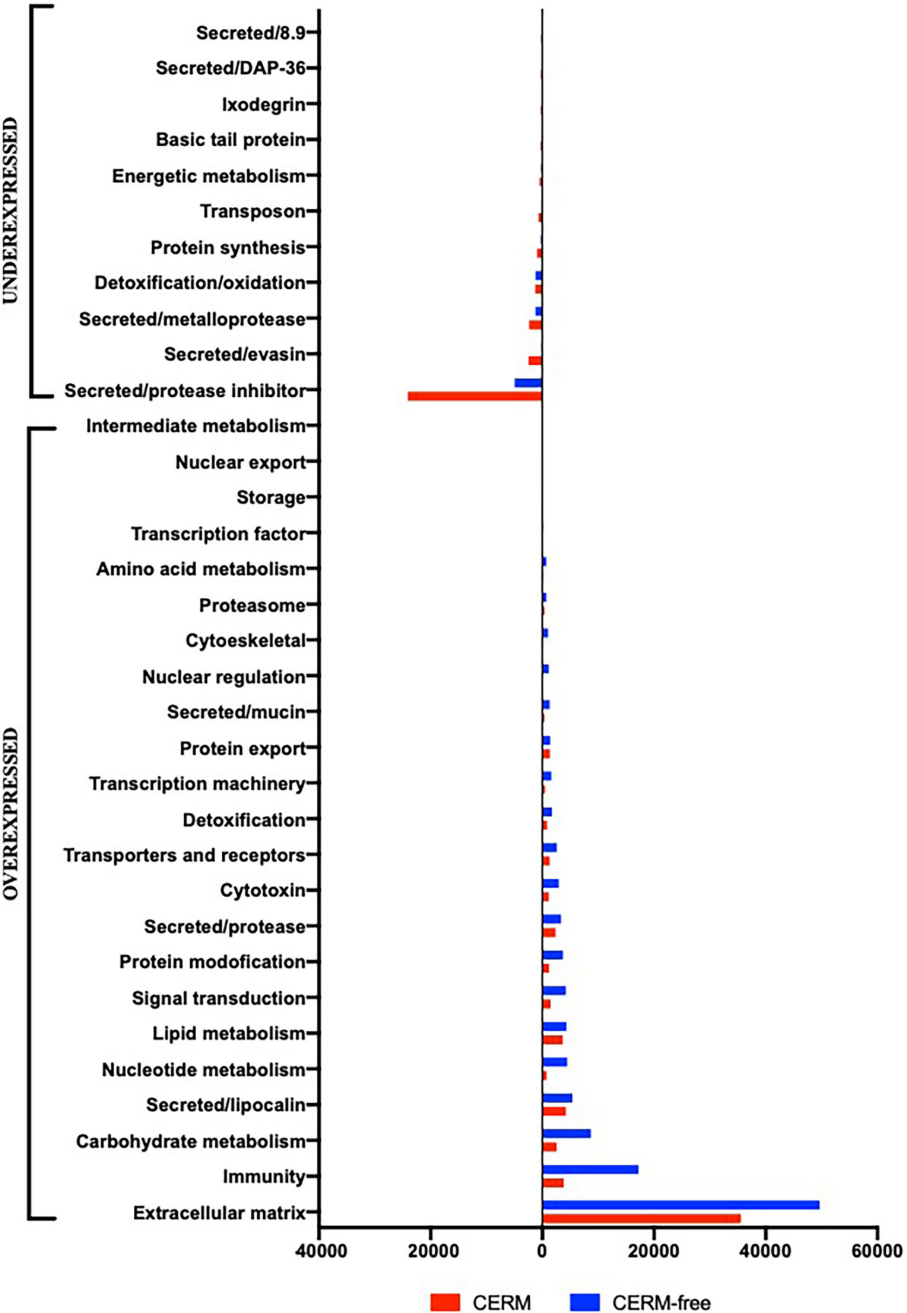
Total TPM (transcripts per million) in each functional category for differentially expressed transcripts underexpressed and overexpressed in CERM (*Coxiella* endosymbiont from *Rhipicephalus microplus*)-free metanymphs. Unknown and Unknown conserved categories were excluded from the analysis.

Using an FDR (False discovery rate) cutoff of 0.05 and analyzing only those transcripts with a CPM value greater than10 in at least 3 libraries, 650 differentially expressed transcripts (DET) were identified (Supplementary Table 1- worksheet: DET). A heat map overview of DET using transcripts per million (TPM) values (Figure 3) demonstrates that the lack of the endosymbiont modulated the host gene expression profile. We then analyzed these identified DETs in two ways. First, we calculated the number of transcripts that were under or overexpressed in CERM-free ticks and binned them into functional categories. Next, we plotted the absolute number of transcripts per million (TPM) for each of these functional categories in CERM and CERM-free metanymphs (Table 2 and Figure 4).

A similar number of transcripts were overexpressed (*n* = 307) or underexpressed (*n* = 343) in CERM-free metanymphs (Table 1). It is relevant that 134 (21%) out of the 650 DETs were functionally categorized either unknown or unknown conserved, corresponding to 45% of the TPM assigned to all DETs. However, these transcripts were not analyzed further as the lack of functional annotation prevented speculations on their biological relevance. The expression profile showed that overexpressed transcripts were assigned to a larger number of functional categories, whereas underexpressed transcripts were largely associated with those involved in blood feeding capacity such as protease inhibitors, evasins, DAP-36 family members, and metalloproteases (Table 2; Figure 4; Bergman et al., 2000; Imamura et al., 2005; Decrem et al., 2008; Liu et al., 2014; Ali et al., 2015; Hayward et al., 2017; Parizi et al., 2020). Remarkably, 57% of underexpressed genes were previously identified in sialotranscriptomes (Ribeiro and Mans, 2020), showing a highly dysregulated salivary gland gene expression profile and strongly suggesting defective salivary function. This finding could explain the blockage in development at the metanymph life stage through an interruption in blood uptake. Also relevant was the dysregulation of transcripts coding for cuticle proteins. Differential expression of transcripts involved in the extracellular matrix could also explain the observed phenotype through the prevention of tick growth and molting. Several categories related to metabolism (carbohydrates, lipids, amino acids, nucleotides, and intermediate metabolism) were overexpressed in the CERM-free group as well as those related to nuclear regulation, immunity, and the extracellular matrix.

**Table 1:** Number of transcripts IDs differentially expressed in CERM (*Coxiella* endosymbiont from *R. microplus*)-free metanymphs grouped into functional categories.

Number of transcripts IDs overexpressed and underexpressed in CERM-free metanymphs grouped into functional categories			
Category	Overexpressed	Underexpressed	Total
Basic tail protein	0	2	2
Cytoeskeletal	18	0	18
Cytotoxin	3	1	4
Detoxification	6	7	13
Detoxification/oxidation	8	12	20
Extracellular matrix	25	20	45
Immunity	8	10	18
Ixodegrin	1	1	2
Amino-acid metabolim	5	1	6
Carbohydrate metabolism	10	11	21
Energetic metabolism	1	5	6
Intermediate metabolism	1	0	1
Lipid metabolism	14	35	49
Nucleotide metabolism	5	5	10
Nuclear export	1	0	1
Nuclear regulation	15	0	15
Protein export	8	6	14
Protein modification	19	3	22
Proteasome	9	2	11
Protein synthesis	3	2	5
Secreted/8.9	0	1	1
Secreted/DAP-36	0	2	2
Secreted/evasin	0	7	7
Secreted/lipocalin	3	22	25
Secreted/metalloprotease	6	29	35
Secreted/mucin	5	2	7
Secreted/protease	6	10	16
Secreted/protease inhibitor	6	35	41
Secreted/unknown conserved	17	41	58
Secreted/unknown	0	7	7
Signal transduction	33	9	42
Storage	1	0	1
Transposon	0	6	6
Transcription factor	4	0	4
Transcription machinery	15	2	17
Transporters and receptors	12	17	29
Unknown conserved	38	26	64
Unknown	1	4	5
Total	307	343	650

**Table 2:** Total TPM (transcripts per million) in each functional category for the differentially expressed transcripts in CERM (*Coxiella* endosymbiont from *R. microplus*)-free metanymphs.

Sum of TPM in CERM and CERM-free metanymphs grouped into functional categories			
Category	CERM	CERM-free	Regulation pattern
Secreted/protease inhibitor	24078.64	4937.82	UNDER
Secreted/evasin	2460.52	158.63	UNDER
Secreted/metalloprotease	2373.48	1219.23	UNDER
Detoxification/oxidation	1278.55	1245.03	UNDER
Protein synthesis	986.93	280.05	UNDER
Transposon	674.3	131.88	UNDER
Energetic metabolism	535.7	200.29	UNDER
Basic tail protein	315.75	52.37	UNDER
Ixodegrin	268.89	88.74	UNDER
Secreted/DAP-36	233.86	17.12	UNDER
Secreted/8.9	204.17	1.32	UNDER
Extracellular matrix	35552.83	49671.50	OVER
Immunity	3841.65	17208.42	OVER
Carbohydrate metabolism	2548.87	8705.11	OVER
Secreted/lipocalin	4208.02	5421.52	OVER
Nucleotide metabolism	766.46	4452.99	OVER
Lipid metabolism	3659.48	4316.08	OVER
Signal transduction	1455.77	4189.93	OVER
Protein modification	1212.22	3697.05	OVER
Secreted/protease	2383.11	3373.91	OVER
Cytotoxin	1150.83	2937.87	OVER
Transporters and receptors	1309.5	2607.53	OVER
Detoxification	835.30	1740.97	OVER
Transcription machinery	508.04	1610.77	OVER
Protein export	1363.44	1410.07	OVER
Secreted/mucin	375.89	1307.63	OVER
Nuclear regulation	165.40	1138.48	OVER
Cytoeskeletal	164,81	1014.25	OVER
Proteasome	379.20	753.54	OVER
Amino-acid metabolism	205.76	737.19	OVER
Transcription factor	57.11	181.83	OVER
Storage	12.79	136.03	OVER
Nuclear export	22.67	102.24	OVER
Intermediate metabolism	2.44	14.42	OVER
Total	95592.38	125061.81	

*UNDER, Underexpressed categories in CERM-free metanymphs; OVER, overexpressed categories in CERM-free metanymphs*.

### DET Involved in Blood Feeding Success

Vertebrate hosts react to skin injury caused by tick bites with the production of molecules with hemostatic, vasoconstrictor, and inflammatory properties, which may disrupt tick feeding, cause developmental arrest, or even result in tick rejection (Nuttall, 2019). As part of host–parasite coevolution, ticks have evolved to secrete molecules in the saliva that counteract the vertebrate host response, increasing the likelihood of feeding success (Ribeiro and Francischetti, 2003; Tabor et al., 2017; Wikel, 2018).

Two transcripts encoding DA-P36 family members were underexpressed in the absence of CERM (Supplementary Table 2), one of which showed the fifth highest fold change value among all underexpressed transcripts (Supplementary Table 1- worksheet: DET). The first member of the DA-P36 family was described in the saliva of *Dermacentor andersoni* (Bergman et al., 2000), followed by the identification of other two homologous molecules in the salivary glands of *Haemaphysalis longicornis* (HL-p36; Nakajima et al., 2005; Konnai et al., 2009) and *Rhipicephalus haemaphysaloides* (RH36; Wang et al., 2017). Among these tick species, DA-P36 proteins inhibited proliferation of host T-lymphocytes, interfering with the host immune response to tick infestation. Since then, transcripts from the DA-P36 family have been identified in salivary glands from several tick species (Anatriello et al., 2010; Ribeiro et al., 2011; Tan et al., 2015; de Castro et al., 2016) and have been shown to be upregulated by feeding on the vertebrate host (Esteves et al., 2017). Moreover, in *R. haemaphysaloides*, vaccination of the vertebrate host with RH36 significantly affected tick feeding success (Wang et al., 2017). This suggests a tripartite species interaction, since CERM can affect tick DA-P36 expression, influence the T-lymphocyte function on the bovine host, and ultimately facilitate tick feeding and infestation.

Evasins have been identified as components of the tick salivary gland and saliva in several tick species (Hayward et al., 2017). Members of this family bind host chemokines, inhibiting the inflammatory response of the host through the recruitment of leukocytes. Seven transcripts encoding for evasins were differentially expressed in our analysis (Supplementary Table 2), all of which were underexpressed in CERM-free metanymphs. In addition, one of the transcripts encoding for an evasin had the ninth highest fold change of all underexpressed transcripts (Supplementary Table 1- worksheet: DET).

The family of lipocalins is comprised of proteins that bind small hydrophobic molecules and participate in many biological processes (Flower et al., 1993). Lipocalins are major components of the saliva of several blood feeding arthropods, including ticks, and perform essential anti-inflammatory and anti-hemostatic roles such as inhibiting platelet aggregation, reducing coagulation, and promoting vasodilation (Andersen et al., 2005; Mans, 2005). In ticks, it has been shown that lipocalins are anti-inflammatory proteins that bind to host histamine at the tick bite site (Valdés, 2014). Histamine is a molecule produced and secreted by basophils and mast cells, which increases the vascular permeability to white blood cells to facilitate the immune response. Sialotranscriptomes from several tick species revealed that lipocalins are overexpressed by blood feeding on a vertebrate host (Peterson et al., 2009; Konnai et al., 2011; Wang et al., 2016; Esteves et al., 2017; Tirloni et al., 2020b). In *R. microplus,* lipocalins were found to be overexpressed in adult female salivary glands and were also abundant in the proteome of saliva (Tirloni et al., 2014a, 2020a). In *Ixodes persulcatus,* the immunization of mice against tick lipocalins delayed the period for nymphs to reach engorgement (Konnai et al., 2011). The same aberrant pattern of feeding on a host was found in adult females of *Amblyomma americanum* when silencing a histamine-binding protein (Aljamali et al., 2003). Also in *A. americanum,* lipocalins were identified as part of the cement cone, which allows hard ticks to feed for long periods, keeping the tick mouthparts anchoring into host skin (Hollmann et al., 2017). From the 25 differentially expressed lipocalins found in our analysis, 24 were classified as salivary due to their similarities with lipocalins identified in tick sialotranscriptomes (Ribeiro and Mans, 2020; Supplementary Table 2), and 22 of these were underexpressed in CERM-free metanymphs. Among those, a salivary lipocalin was the second most underexpressed transcript in CERM-free metanymphs (Supplementary Table 1- worksheet: DET).

Expression of members of serine and cysteine protease inhibitor superfamilies were significantly altered in CERM-free metanymphs (Supplementary Table 1- worksheet: DET). From the 41 differentially expressed protease inhibitors, 27 were identified in tick sialotranscriptomes (Supplementary Table 2). Among them, 17 were classified as serine inhibitors containing Kunitz-type domains and 14 of those were underexpressed in CERM-free metanymphs. Among other functions, Kunitz-type inhibitors are anti-hemostatic agents that block host coagulation and/or platelet aggregation (Corral-Rodríguez et al., 2009; Parizi et al., 2018). Silencing of a salivary Kunitz-type inhibitor gene impaired *Ixodes ricinus* blood feeding, resulting in decreased tick weight (Liu et al., 2014). Ten transcripts belonging to the trypsin inhibitor-like family (TIL) were among the DET and all of them were underexpressed in CERM-free metanymphs. Out of those, six were identified in tick sialotranscriptomes (Ribeiro and Mans, 2020; Supplementary Table 2). In *R. microplus,* transcripts for TIL domain-containing peptides were overexpressed in salivary glands (Tirloni et al., 2020a). In ticks, members of the TIL family showed anti-elastase and antimicrobial activities (Fogaça et al., 2006; Sasaki et al., 2008). Two salivary serpins were overexpressed in CERM-free metanymphs (Supplementary Table 2). Serpins are inhibitors of serine and cysteine proteases and are involved in a diverse range of tick biological functions, including modulation of host proteases to facilitate the feeding process (Chmelař et al., 2017). In *R. microplus,* several serpins were expressed during blood feeding (Tirloni et al., 2014b). Vaccination of vertebrate hosts with a salivary serpin from *H. longicornis* resulted in increased mortality in nymphal and adult ticks (Imamura et al., 2005). Two members of cystatin family, another group of cysteine protease inhibitors, were among the DET underexpressed in CERM-free ticks (Supplementary Table 2). Both transcripts have previously been identified in tick sialotranscriptomes. Cystatins have been found in the salivary glands, saliva, and in the midgut of several tick species. These proteins perform anti-inflammatory and immunosuppressant roles, in addition to regulating blood digestion (Kotsyfakis et al., 2006; Lu et al., 2020). Inhibition of cystatin family members by RNA interference or through feeding on a vertebrate host immunized with recombinant cystatin showed a negative impact on tick feeding success, with decreased attachment rate and engorgement weight observed (Kotsyfakis et al., 2008; Salát et al., 2010; Parizi et al., 2020).

Finally, from the 35 DET coding for metalloproteases, 32 were identified in tick sialotranscriptomes (Ribeiro and Mans, 2020; Supplementary Table 1- worksheet: DET). From those, 27 were underexpressed in CERM-free metanymphs (Supplementary Table 2) and 19 out of those were members of the M12 family, which are similar to the hemorrhagic proteases of snake venom. In *R. microplus* M12 metalloproteases were found to be overexpressed in adult female salivary glands (Tirloni et al., 2020a) and in *I. scapularis* a metalloprotease from the M12 family displayed fibrin(ogen)lytic and gelatinase activities within the saliva (Francischetti et al., 2003). Vaccination against a *R. microplus* M12 salivary metalloprotease protected its vertebrate host from tick infestation by interfering with the completion of the blood meal (Ali et al., 2015). A similar effect was observed when a M12 metalloprotease from *I. ricinus* was used as vaccinal antigen, causing a reduction in tick feeding time and weight gain (Decrem et al., 2008).

### DET Involved in Extracellular Matrix Formation and Molting

The integument of arthropods is a multi-layered cuticle secreted by the epidermis (Hackman, 1982). It forms the exoskeleton, which protects against external agents and prevents water loss. The exoskeleton is a limiting factor to expand and, as a consequence, arthropods need to molt in order to grow. However, ticks are exceptional as they are capable of increasing their body weight several fold before ecdysis (Dillinger and Kesel, 2002). Integument expansion during host blood intake to accommodate the enlarged gut occurs due to their unique cuticle structural extensibility (Hackman, 1982). Arthropod cuticles are made of several proteins, each with a unique temporal expression profile. Their expression is activated by distinct sets of transcription factors that participate in the ecdysone signaling cascade (Nijhout et al., 2014). It has been demonstrated in *I. ricinus* that the structure of the cuticle changes during the blood feeding, which could be explained by a time-dependent change in gene expression (Dillinger and Kesel, 2002). Therefore, disruption of the proper schedule of cuticle gene expression is likely to have a large impact on cuticle structure, resulting in consequences to tick engorgement, and ecdysis. Furthermore, it has been shown that treating *Rhipicephalus sanguineus* with fluazuron, an inhibitor of chitin synthesis, was responsible for disorganization of engorged nymph cuticle, which prevented molting into the adult life stage (De Oliveira et al., 2014). Among the extracellular matrix transcripts, four were assigned as cuticle proteins, two of these were underexpressed, and the other two were overexpressed in the CERM-free metanymph (Supplementary Table 2). One explanation for this is that altered tick development could be due to the disordered temporal expression profile of cuticle proteins in CERM-free metanymphs. This could lead to underexpression of cuticle proteins that should be expressed at the metanymph stage and overexpression of proteins that, in a regular developmental program, would be expressed in a different temporal pattern. Another relevant dysregulation in the transcription of genes involved with extracellular matrix formation and molting was the overexpression of four chitinases and the 20-hydroxy-ecdysone receptor in CERM-free ticks (Supplementary Table 2). 20-hydroxy-ecdysone is a hormone that controls molting in arthropods (Dubrovsky, 2005). As observed in *Bombyx mori*, during the molting process chitinases are induced by ecdysteroids to degrade the older chitin (Kimura, 1973). Our results suggest that, in the absence of CERM, tick chitinases as well as the 20-hydroxy-ecdysone receptor may be overexpressed as a compensatory attempt to force the metanymph to molt into the adult life stage. As observed in the CERM-*R. microplus* interaction, other arthropod-symbiont relationships impact the host cuticle formation (Hirota et al., 2017). Aposymbiotic *Oryzaephilus surinamensis*, the saw-toothed grain beetle, presented an alteration on the cuticle’s color, showing that the endosymbiont contributes to the host’s cuticle formation (Hirota et al., 2017). Similarly, the endosymbiont lineage *Nardonella* found in weevils showed to have an impact on the host cuticle’s formation (Anbutsu et al., 2017). Suppression of *Nardonella* resulted in the emergence of reddish and soft insects. This phenotype was correlated with low titer of tyrosine, which can only be synthesized in the presence of the endosymbiont. In the turtle ant Cephalotes, the gut symbiotic community contributes to the formation of chitin in the host insect cuticle through nitrogen enrichment (Duplais et al., 2021). However, as vertebrate blood has a very high protein content (about 85% of blood dry weight), it seems unlikely that a similar mechanism would apply for the tick/CERM symbiosis.

### Overexpressed Functional Classes in Response to the Absence of CERM

Although the most striking consequence of the lack of the endosymbiont was the underexpression of transcripts involved in the acquisition of blood feeding capacity, the elimination of CERM also resulted in the overexpression of some transcripts in the *R. microplus* metanymphs (Table 2 and Figure 4). All functional categories related to metabolism were overexpressed. Moreover, transcripts associated with nuclear regulation, immunity, and extracellular matrix were overexpressed in the CERM-free group. The overexpression of these functional categories could also indicate a physiological compensation for the reduced flux in metabolic pathways due to impaired blood acquisition and digestion. Recently, the concept of “sialome switching” has been proposed for different tick species showing that the proteomic or transcriptional profile of the tick salivary glands switches at intervals as a mechanism to evade the vertebrate host immune response against tick infestation (Karim and Ribeiro, 2015; Perner et al., 2018; Tirloni et al., 2020b). Also, it was observed that ticks can secrete saliva with different protein profiles when exposed to different host species (Tirloni et al., 2017). Both observations suggest a high degree of variability for gene expression, conferring flexibility in tick feeding behavior. Although we have not performed time-course studies, it is possible that alterations in the CERM-free group represent a dysregulated temporal expression profile, resulting in the presence of transcripts that should be expressed at different time points of development. Thus, the disruption of a coordinated time-expression course of salivary transcripts could negatively interfere with the blood feeding process. Though we can only speculate on the reason why several functional categories are overexpressed, it is likely that the overall dysregulation in gene expression contributed to the interruption of feeding and proper development to adult stage.

### Physiological Role of Tick Endosymbionts in Tick Feeding and Growth

As observed for CERM-free ticks, the elimination of the endosymbiont *Francisella* sp. from its tick host, *Ornithodoros moubata,* led to an interruption of molting to the following life stage (Duron et al., 2018). In that case, the blockage in development was due to an interruption in blood intake. Remarkably, the ticks showed normal blood ingestion and molting when B vitamins were added to their diet. As exclusively hematophagous parasites, ticks rely on host blood to obtain all nutrients needed for development. Though blood is rich in lipids and proteins, it is a relatively poor source of vitamins, and it has been shown that ticks endosymbionts can supply ticks with the vitamins they require. In addition to *Francisella* sp., the genomes of several tick endosymbionts, including CERM, encode genes for the biosynthesis pathways of vitamins of complex B and cofactors (Smith et al., 2015; Guizzo et al., 2017; Duron et al., 2018). Both *Francisella* and CERM have the ability to produce biotin, riboflavin, folic acid, CoA, and FAD. Therefore, it is possible that the interruption in development of CERM-free *R. microplus* was due to the insufficient supply of B vitamins and cofactors that are critical for several key metabolic enzymes, likely including those involved in blood feeding success and the molting process. We have found an overexpression of ornithine decarboxylase in CERM-free ticks. This enzyme has pyridoxal phosphate (B6) as a coenzyme which is one of the B vitamins synthesized by CERM (Guizzo et al., 2017). We speculate that ticks lacking CERM overexpress ornithine decarboxylase as a regulatory response to synthesize compounds that are absent due to the lack of these vitamin. Nevertheless, the absence of transcriptional control of other enzymes, of which vitamins are cofactors, does not necessarily argue against a role for vitamin supply in the regulation of transcriptional activity but could indicate that post-transcriptional mechanisms may be involved in the impact of vitamins on metabolism. Further metabolomics analyses may reveal nutrient-metabolism interactions, correlating the absence of CERM with the absence of compounds derived from the metabolism of B vitamins.

Recently, *Coxiella* from *H. longicornis* (CHl) was associated with regulation of tick feeding and subsequent growth by influencing serotonin biosynthesis through chorismate, a tryptophan precursor (Zhong et al., 2021). Serotonin has been shown to be one of the neural-related molecules that regulates salivary gland function during and after feeding in several species. We could not find any relevant underexpression of transcripts involved in amino-acid metabolism as was shown for *H. longicornis*. While both studies showed impaired blood feeding resulting from the reduction in *Coxiella* levels, the profile described here seems to involve a larger collection of genes, including a number of transcripts of unknown function. The differences between these two transcriptomic analyses could explain these distinct findings as (i) *R. microplus* are one-host ticks; (ii) the ticks analyzed in this study were from a later stage of development than those described by Zhong et al. (2021); and (iii) the ticks in our study were completely CERM-free, in contrast to the study focused on *H. longicornis*. Despite the differences in these studies, their disparate observations may in fact be an indication of the complexity of host-symbiont interactions and their impact on tick physiology.

## Conclusion

Our previous work has shown that *R. microplus* ticks with significantly reduced levels of CERM had their development blocked at the metanymph stage (Guizzo et al., 2017). Here, we described *R. microplus* transcriptional changes in CERM-free metanymphs. It has been shown that tick-borne pathogens influence host gene expression (Zivkovic et al., 2010; Mercado-Curiel et al., 2011; Liu and Bonnet, 2014; Rosa et al., 2016; Capelli-Peixoto et al., 2017; Carvajal-de la Fuente et al., 2018; Kim et al., 2021). However, interaction of non-pathogenic microbes with their tick hosts remains insufficiently explored.

**Figure 5:**
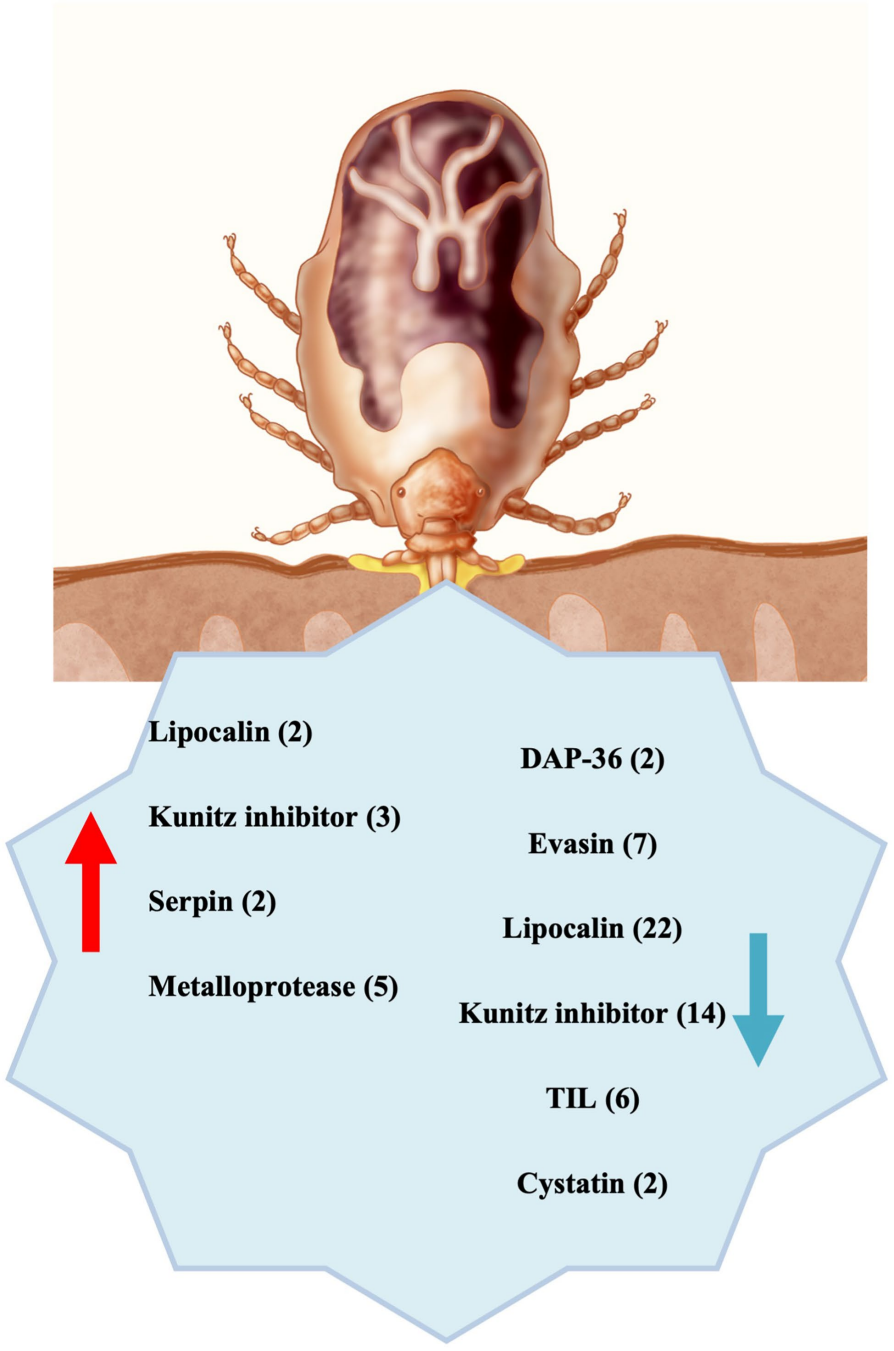
Summary scheme: Differentially expressed transcripts underexpressed (blue arrow) and overexpressed (red arrow) in CERM (*Coxiella* endosymbiont from *Rhipicephalus microplus*)-free metanymph were significantly involved with blood intake, suggesting that the endosymbiont is essential for building the blood feeding capacity during development. The number of transcripts from each category is indicated in the parentheses.

The results from this study demonstrate that several functional categories of genes are highly altered in the absence of the symbiont. We highlight the dramatic underexpression of salivary proteins, which are essential for the blood feeding success (Figure 5). Moreover, the dysregulation of cuticle protein expression in CERM-free metanymphs could compromise the ability of the tick to expand during blood feeding, thereby inhibiting molting. All together, these two components could explain the blockage in development at the metanymph stage, revealing intriguing physiological aspects of the interaction between symbiont and tick host.

## Data Availability Statement

The datasets presented in this study can be found in online repositories. The names of the repository/repositories and accession number(s) can be found in the article/Supplementary Material.

## Ethics Statement

All animal care and experimental protocols were conducted following the guidelines of the institutional care and use committee (Ethics Committee on Animal Experimentation of the Universidade Federal do Rio Grande do Sul) and were approved under registry 28108 and 14403.

## Author Contributions

MG, PO, and IS conceived and designed the study. MG, LP, and LG generated the data. LT, MF, SG, and GB acquired and analyzed the data. MG and LT interpreted the data. MG wrote the first draft. PO, IS, and LT substantially revised subsequent drafts. All authors edited the manuscript, commented on the text, and approved the final version of the manuscript.

## Funding

This work was supported by Conselho Nacional de Desenvolvimento Tecnológico—CNPq, Coordenação de Aperfeiçoamento de Pessoal de Nível Superior—CAPES and INCT–Entomologia Molecular, Brazil. LT was supported by the Intramural Research Program of the National Institute of Allergy and Infectious Diseases (Z01 AI001337-01). This work utilized the computational resources of the NIH HPC Biowulf cluster (http://hpc.nih.gov).

## Conflict of Interest

The authors declare that the research was conducted in the absence of any commercial or financial relationships that could be construed as a potential conflict of interest.

## Publisher’s Note

All claims expressed in this article are solely those of the authors and do not necessarily represent those of their affiliated organizations, or those of the publisher, the editors and the reviewers. Any product that may be evaluated in this article, or claim that may be made by its manufacturer, is not guaranteed or endorsed by the publisher.

## Footnotes

^1^ https://www.bioinformatics.babraham.ac.uk/projects/trim_galore/

^2^ https://www.cbs.dtu.dk/services/

^3^ https://geneontology.org/

^4^ ftp://ftp.ncbi.nih.gov/pub/mmdb/cdd/little_endian/

^5^ https://mycocosm.jgi.doe.gov/help/kogbrowser.jsf

^6^ https://www.ncbi.nlm.nih.gov/genbank/

^7^ https://www.ebi.ac.uk/uniprot/

## Supplementary Material

Supplementary Table 1 Hyperlinked spreadsheet containing the annotated assembly of the *Rhipicephalus microplus* metanymph transcriptome can be found at: https://proj-bip-prod-publicread.s3.amazonaws.com/transcriptome/R_microplus_Coxiella/Rm-Coxiella.zip.

Supplementary Table 2 Differentially expressed transcripts in CERM-free metanymph involved in blood feeding capacity and extracellular matrix formation and molting.
