# Supplementary material for: Coxiella Endosymbiont of Rhipicephalus microplus Modulates Tick Physiology With a Major Impact in Blood Feeding Capacity
Source: Front Microbiol. 2022 May 3;13:868575. doi: 10.3389/fmicb.2022.868575 (PMC9111531; doi:10.3389/fmicb.2022.868575)
Supplement: Supplementary Table 1 — Hyperlinked spreadsheet containing the annotated assembly of the Rhipicephalus microplus metanymph transcriptome can be found at: https://proj-bip-prod-publicread.s3.amazonaws.com/transcriptome/R_microplus_Coxiella/Rm-Coxiella.zip. [file Table_1.DOCX]

| **Number of transcripts IDs overexpressed and underexpressed in CERM-free metanymphs grouped into functional categories** | | | |
| --- | --- | --- | --- |
| **Category** | **Overexpressed** | **Underexpressed** | **Total** |
| Basic tail protein | 0 | 2 | 2 |
| Cytoeskeletal | 18 | 0 | 18 |
| Cytotoxin | 3 | 1 | 4 |
| Detoxification | 6 | 7 | 13 |
| Detoxification/oxidation | 8 | 12 | 20 |
| Extracellular matrix | 25 | 20 | 45 |
| Immunity | 8 | 10 | 18 |
| Ixodegrin | 1 | 1 | 2 |
| Amino acid metabolim | 5 | 1 | 6 |
| Carbohydrate metabolism | 10 | 11 | 21 |
| Energetic metabolism | 1 | 5 | 6 |
| Intermediate metabolism | 1 | 0 | 1 |
| Lipid metabolism | 14 | 35 | 49 |
| Nucleotide metabolism | 5 | 5 | 10 |
| Nuclear export | 1 | 0 | 1 |
| Nuclear regulation | 15 | 0 | 15 |
| Protein export | 8 | 6 | 14 |
| Protein modification | 19 | 3 | 22 |
| Proteasome | 9 | 2 | 11 |
| Protein synthesis | 3 | 2 | 5 |
| Secreted/8.9 | 0 | 1 | 1 |
| Secreted/DAP-36 | 0 | 2 | 2 |
| Secreted/evasin | 0 | 7 | 7 |
| Secreted/lipocalin | 3 | 22 | 25 |
| Secreted/metalloprotease | 6 | 29 | 35 |
| Secreted/mucin | 5 | 2 | 7 |
| Secreted/protease | 6 | 10 | 16 |
| Secreted/protease inhibitor | 6 | 35 | 41 |
| Secreted/unknown conserved | 17 | 41 | 58 |
| Secreted/unknown | 0 | 7 | 7 |
| Signal transduction | 33 | 9 | 42 |
| Storage | 1 | 0 | 1 |
| Transposon | 0 | 6 | 6 |
| Transcription factor | 4 | 0 | 4 |
| Transcription machinery | 15 | 2 | 17 |
| Transporters and receptors | 12 | 17 | 29 |
| Unknown conserved | 38 | 26 | 64 |
| Unknown | 1 | 4 | 5 |
|  |  |  |  |
| **Total** | **307** | **343** | **650** |
