# Supplementary material for: Coxiella Endosymbiont of Rhipicephalus microplus Modulates Tick Physiology With a Major Impact in Blood Feeding Capacity
Source: Front Microbiol. 2022 May 3;13:868575. doi: 10.3389/fmicb.2022.868575 (PMC9111531; doi:10.3389/fmicb.2022.868575)
Supplement: Supplementary Table 2 — Differentially expressed transcripts in CERM-free metanymph involved in blood feeding capacity and extracellular matrix formation and molting. [file Table_2.DOCX]

| **Sum of TPM in CERM and CERM-free metanymphs grouped into functional categories** | | | |
| --- | --- | --- | --- |
| Category | **CERM** | **CERM-free** | **Regulation pattern** |
| Secreted/protease inhibitor | 24078.64 | 4937.82 | UNDER |
| Secreted/evasin | 2460.52 | 158.63 | UNDER |
| Secreted/metalloprotease | 2373.48 | 1219.23 | UNDER |
| Detoxification/oxidation | 1278.55 | 1245.03 | UNDER |
| Protein synthesis | 986.93 | 280.05 | UNDER |
| Transposon | 674.3 | 131.88 | UNDER |
| Energetic metabolism | 535.7 | 200.29 | UNDER |
| Basic tail protein | 315.75 | 52.37 | UNDER |
| Ixodegrin | 268.89 | 88.74 | UNDER |
| Secreted/DAP-36 | 233.86 | 17.12 | UNDER |
| Secreted/8.9 | 204.17 | 1.32 | UNDER |
| Extracellular matrix | 35552.83 | 49671.50 | OVER |
| Immunity | 3841.65 | 17208.42 | OVER |
| Carbohydrate metabolism | 2548.87 | 8705.11 | OVER |
| Secreted/lipocalin | 4208.02 | 5421.52 | OVER |
| Nucleotide metabolism | 766.46 | 4452.99 | OVER |
| Lipid metabolism | 3659.48 | 4316.08 | OVER |
| Signal transduction | 1455.77 | 4189.93 | OVER |
| Protein modification | 1212.22 | 3697.05 | OVER |
| Secreted/protease | 2383.11 | 3373.91 | OVER |
| Cytotoxin | 1150.83 | 2937.87 | OVER |
| Transporters and receptors | 1309.5 | 2607.53 | OVER |
| Detoxification | 835.30 | 1740.97 | OVER |
| Transcription machinery | 508.04 | 1610.77 | OVER |
| Protein export | 1363.44 | 1410.07 | OVER |
| Secreted/mucin | 375.89 | 1307.63 | OVER |
| Nuclear regulation | 165.40 | 1138.48 | OVER |
| Cytoeskeletal | 164,81 | 1014.25 | OVER |
| Proteasome | 379.20 | 753.54 | OVER |
| Amino acid metabolism | 205.76 | 737.19 | OVER |
| Transcription factor | 57.11 | 181.83 | OVER |
| Storage | 12.79 | 136.03 | OVER |
| Nuclear export | 22.67 | 102.24 | OVER |
| Intermediate metabolism | 2.44 | 14.42 | OVER |
|  |  |  |  |
| **Total** | 95592.38 | 125061.81 |  |
